# Effectiveness of Virtual Reality–Based Well-Being Interventions for Stress Reduction in Young Adults: Systematic Review

**DOI:** 10.2196/52186

**Published:** 2024-03-29

**Authors:** Joy Xu, Areej Khanotia, Shmuel Juni, Josephine Ku, Hana Sami, Vallen Lin, Roberta Walterson, Evelyn Payne, Helen Jo, Parmin Rahimpoor-Marnani

**Affiliations:** 1 David Geffen School of Medicine at UCLA Los Angeles, CA United States; 2 Faculty of Science University of Ottawa Ottawa, ON Canada; 3 Faculty of Arts and Science University of Toronto Toronto, ON Canada; 4 Faculty of Health Sciences McMaster University Hamilton, ON Canada; 5 Faculty of Science York University Toronto, ON Canada

**Keywords:** well-being, well-being, virtual reality, VR, stress, nature, academic, student, intervention, young adults, teens, adolescent, stressors, stress management, systematic review, accessible, accessibility, students, affordable

## Abstract

**Background:**

Adolescents can be especially vulnerable to various stressors as they are still in their formative years and transitioning into adulthood. Hence, it is important for them to have effective stress management strategies.

**Objective:**

This systematic review investigates current well-being interventions that are aimed at reducing stress among young adults. In particular, interventions using the medium of virtual reality (VR) are explored.

**Methods:**

This mixed methods systematic review follows the PRISMA-P (Preferred Reporting Items for Systematic Reviews and Meta-Analyses Protocols) guidelines, and papers were gathered from databases such as PsycINFO, PubMed, Science Direct, Web of Science, OpenGrey, and Edutopia. Predetermined criteria and specific keywords were used to search for the papers. Search results were screened and extracted with all article screening or extraction delegated among all authors. Any disagreements after reconciliation were settled by a third author. The quality and risk of bias of included studies were assessed using the GRADE (Grading of Recommendations Assessment, Development, and Evaluation) Tool for Quantitative Studies. Studies were analyzed qualitatively.

**Results:**

In total, 20 studies were included, and qualitative analysis was performed to evaluate the effectiveness of VR-based interventions in 3 domains: nature, stress, and academics.

**Conclusions:**

Studies using VR interventions, overall, promoted a reduction in stress and an increase in well-being. The findings suggest that VR may serve as an accessible and affordable medium of stress reduction for students and young adults. Larger sample sizes, and a greater number of included studies, may be required in future directions.

## Introduction

The COVID-19 pandemic has impacted millions of lives across the world and provided a spotlight on systemic disparities including present limitations to well-being and accessibility for support. The reverberations of this global phenomenon have reconciled greater focus on adolescents and young adults in particular, as their formative years were inevitably impacted by social distancing. The crucial years of their education were faced with obstacles in course delivery, academic opportunities, and social spheres. Furthermore, stresses were compounded with other hardships such as economic setbacks, limited socialization, and more issues that cumulatively burden one’s well-being, especially in the transition into adulthood. Thus, there has been significant effort to identify potential targets for interventions through research. Broadly, interventions aim to help study some change in individual experiences through strategies and processes after a systematic modification [1]. The main objective is to measure the effect of a process or program on certain situations [1]. In this review, virtual reality (VR) interventions refer to programs or treatments that target one or more determinants of health using a VR headset, which displays a visual environment. This review refers to the Canadian Index’s definition of Well-being stated as “The presence of the highest possible quality of life in its full breadth of expression focused on but not necessarily exclusive to good living standards, robust health, a sustainable environment, vital communities, an educated populace, balanced time use, …” [2]. Thus, some aspects of an individual’s well-being can be measured through the extent to which an individual is stressed. Stress can be understood as a “psychophysiological response” to some form of danger and involves biological components including nervous and hormonal responses to stimuli [3].

This systematic review questions the effectiveness of VR interventions in reducing stress and promoting well-being in students and young adults. Currently, mindfulness-based interventions (MBIs) through digital or computerized means are common, such as mindfulness-based stress reduction, group mindfulness-based intervention, and self-direct mindfulness-based intervention through digital delivery. There is evidence that these interventions have significant improvement in regulating emotion and mindfulness [4]. Similarly, there has been progressive growth in present research demonstrating the efficacy of VR interventions for well-being. VR can be defined as an artificial, 3D digital environment that a user experiences through a computer headset [5]. Although many reviews in the literature summarize the efficacy or need for MBIs delivered on a computer or digital program, there remains a need for a comprehensive review to specifically assess the impact of VR-based interventions on stress and mental well-being among students and young adults.

Currently, the use of nature-based settings alone was found to improve mood to being “good” and “calm” in older adults, without the need for an MBI curriculum [6]. Thus, the papers included in this systematic review use auditory, visual, and even olfactory aids to simulate an MBI to reduce stress and increase mindfulness in young adults, such as integrating MBI techniques within VR or similar tools or resources.

This systematic review aims to understand the effectiveness of current well-being VR interventions in reducing stress among young adults. Due to the nature of learning in the 21st century relying heavily on digital resources, implementing digital tactics is imperative to combating these new sources of stress. In an era where Generation Z (the generation born between 1997 and 2012) is more stressed than previous generations and most are experiencing burnout [7], having digital resources allows for immediate and low-maintenance aid.

## Methods

A diverse range of literature was assessed according to the inclusion and exclusion criteria, which were determined prior to the search. These criteria qualified the contextual and scientific relevance of the data by developing a standardized expectation for the literature’s content and experimental purpose. The PRISMA-P (Preferred Reporting Items for Systematic Review and Meta-Analysis Protocols; Multimedia Appendix 1 [8]) guideline was used for this qualitative review. There was an inclusion of gray literature in accordance with Assessing the Methodological Quality of Systematic Reviews-2 (AMSTAR-2), as this body of research encompasses material produced outside of traditional commercial or academic publications to reduce publication bias (eg, government databases and preprints). Papers published between 1980 and 2022, which were randomized controlled trials, were gathered from PsycINFO, PubMed, Science Direct, Web of Science, Open Grey, and Edutopia through an initial screening. A specific keyword search was used to gather these papers including the following keywords from each of the search platforms (“wellbeing” OR “well-being” AND “student” AND “virtual” AND “reality” AND “quantitative” AND “intervention”). Duplicates and papers not matching the screening questionnaire were eliminated.

This review considers young adults to be within the age range of 15-40 years. All studies included are published in English. Included studies focused on the analysis of diverse student demographics in the scope of university programs, socioeconomic status, and culture for a robust analysis of student well-being. The interventions under study in this paper include any VR-based environments that can be accessed by a participant through a VR headset, where concepts can be incorporated into a mobile app. Furthermore, stress reduction interventions that use either physiological or psychological questionnaires were included. Exclusion criteria included any research that generated qualitative data and studies published by organizations with conflicts of interest. Studies with interventions geared toward individuals with psychological illnesses and preexisting psychological illnesses were also excluded to reduce confounding factors and external influences or experiences that may uniquely affect the study outcomes in these demographics.

Data for this study were collected and screened using the Covidence software (Veritas Health Innovation). Multiple authors (JX, AK, SJ, JK, HS, VL, RW, EP, HJ, and PRM) screened a collection of research for relevant studies in 2 steps: abstract screening and full-text screening. From a dropdown menu, authors selected a reason for excluding an article (ie, wrong outcomes: Where the paper reports on findings not in line with the research question of systematic reviews, such as pilot studies, no quantitative data, and biological markers of stress). Two authors were assigned to each study. The authors worked independently and reconciled any disagreements and discrepancies after each step. Reconciliation proceeded by a third author who reevaluated the inclusion and exclusion criteria of the study based on the screening or extraction tools independently of the 2 other authors. Following screening, the authors extracted relevant information to be used within this review, which included methods of study design, interventions, subject information, and outcomes. EndNote (McMaster University) was used to combine all studies found and to remove any duplicates. GRADE (Grading of Recommendations Assessment, Development, and Evaluation) was used to assess the quality of the studies. GRADE consists of 4 levels of quality: very low, low, moderate, and high. The 2 authors independently evaluated the included studies using the GRADE methodology. When discrepancies arose regarding the assessed quality levels, the said authors collaborated to resolve them, ultimately reaching a consensus on the final quality rating for those studies.

In this case, all studies that did not appropriately follow the inclusion criteria were marked as wrong outcomes, study interventions, and so forth.

## Results

**Figure 1:**
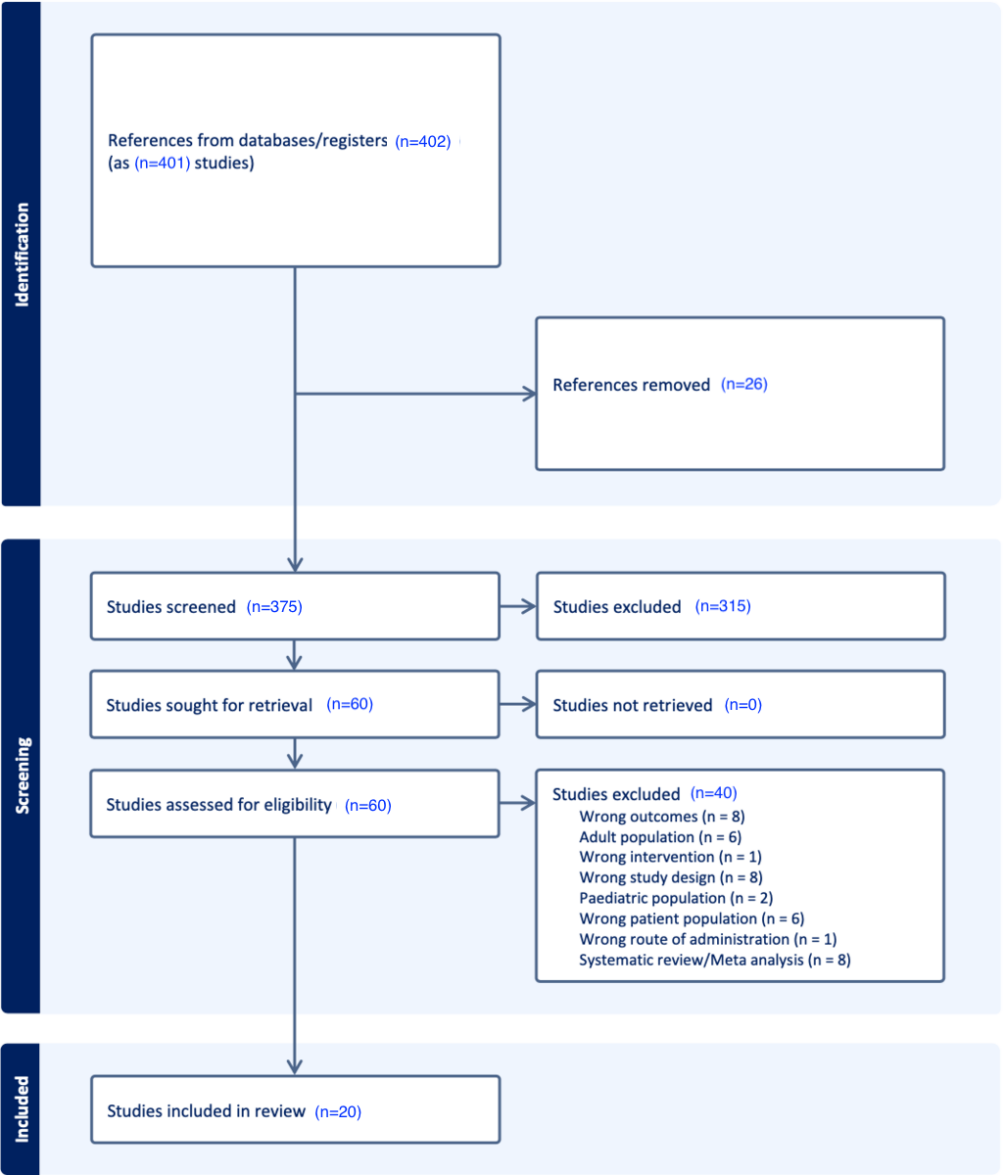
Structural diagram presenting screening and included studies.

The study selection and screening process is outlined in Figure 1. In total, 401 papers were identified from the database search, out of which 20 were included studies. Among these included studies, 11 were randomized controlled trials and 4 were experimental designs. Both the published and gray literature papers went through abstract screening and full-text screening. After the abstract screening, 60 papers remained in total. After full-text screening, 20 papers were left. Through full-text screening, 20 papers remained with categories of nature, stress, and academic contexts within the scope of well-being within the study designs. Studies primarily focused on undergraduate students with included studies encompassing Canada, Ireland, the United States, China, Italy, Europe, Spain, and Russia. Table 1 shows an overview of diverse studies examining the impact of VR environments, with a focus on the nature setting. Key details include authors, publication year, methods, participant populations, interventions, and main findings related to positive and negative affect schedule scores. The studies demonstrate the varied effects of nature-based VR on well-being across different populations. Table 2 presents a synthesis of various studies investigating the effectiveness of VR interventions on stress management and well-being. The studies, conducted using randomized controlled trials and between-subject designs, explore the impact of VR interventions on stress reduction, emotional regulation, relaxation, and positive affect across diverse participant populations. Table 3 provides a comprehensive overview of studies interventions for stress management and well-being among student populations. It outlines various research methodologies, participant demographics, intervention types, and significant findings, offering insights into the use of VR within educational contexts to promote mental health.

**Table 1:** Summary of VR^a^ environment studies (nature).

Authors and year of publication	Methods	Population	Interventions	Main findings
Valtchanov and Ellard (2010) [9]	Randomized controlled trial	Undergraduate students aged 18-26 years from the University of Waterloo, Canada Participants who can read or write English fluently, do not experience “seizures, vertigo, or motion sickness” or severe visual impairment	3 VR settings: Nature (tropical scenery) Geometric (assorted 3-dimensional shapes) Urban (to-scale model of Shibuya station, Tokyo, Japan)	Mean positive affect scores in terms of ZIPERS scores were only significant in the Nature group post immersion in VR, with a mean of 2.21 (SD 0.71) prior to VR compared to 3.03 (SD 0.98) postintervention (*P*<.001). Both geometric and urban VR settings had no significant impact on improving positive effects
O’Meara et al (2020) [10]	Randomized controlled trial	18 years old or older Experience high test anxiety and no treatment Control: low or healthy level of test anxiety From University College Cork, Ireland	2 VR settings: Urban environment Nature environment	Only the high anxiety group of students significantly benefited from the nature VR intervention within the 4-minute session (*P*=.02). The authors conclude that simulated exposure to nature can therefore reduce negative affect, thereby reducing test anxiety. Conversely, virtual exposure to nature settings did not significantly improve test scores
Browning et al (2020) [11]	Randomized controlled trial	18-27 years old, mean age 20 years University students, from the University of Illinois at Urbana-Champaign, the United States Excluded if: Diagnosed mood disorder, treated for mental illnesses, hearing impairments, use of alcohol or prescription drugs not normally taken in 24 hours. Intense physical activity in 24 hours	Control: sit in front of a blank wall Outdoor group: Forest VR group: same setting as forest group	Both VR and outdoor interventions have positive and significant results (*P*<.001), indicating high positive effect scores. All 3 conditions reveal a significant decrease in negative affect values (outdoor group *P*=.034; control group *P*<.001, VR *P*=.03). Only the outdoor group reported a statistically significant increase in positive effect for mood effects (*P*=.044)
Gao et al (2019) [12]	Randomized controlled trial	120 Chinese college students with mean age of 20.7 years Myopia degrees <800	6 different VR environment interventions: Gray Blue Open green Partly open green Partly closed green Closed green	Partly open green had the most impact on reducing negative mood, while closed green had the least. Additionally, student preference for environments revealed that blue was most preferred, with gray and closed green as least preferred. A strong positive correlation is revealed between preference for a given environment and positive mood improvement

^a^VR: virtual reality.

**Table 2:** Overview of studies on VR^a^ interventions for stress management and well-being.

Authors and year of publication	Methods	Population	Interventions	Main findings
Villani and Riva (2012) [13]	Between-subject design	36 Italian participants were seen to be on or past the higher quartile on stress	6-session stress management intervention VR experimental: used “ESCAPE” VR Video experimental group Audio experimental group	Participants were able to reduce their heart rate across conditions and with time. This was specifically seen in the VR condition, where they were more able to reduce their heart rate which in turn helped their emotional state
Wayment et al (2015) [14]	Randomized controlled trial	32 female first-year undergraduate students	Audio recording describing the 4 characteristics of the quiet ego Audio recording with VR of a park scene Control group: read nature magazines	The VR with the audio Quiet Ego Contemplation reduced the degree to which participants felt “in the moment” as compared to the audio-only group (*P*<.05)
Villani and Riva (2008) [15]	Randomized controlled trial	36 individuals who were students or office workers from Milan	Nature VR (park, waterfall, river, garden, etc) Nature video (park, waterfall, river, garden, etc) Relaxing audio	VR, video, and audio of nature settings can help induce relaxation and help in stress management, increase positive emotions, enhance self-awareness, and contribute to emotional regulation. Of the 3 interventions, the VR intervention was seen to have the greatest psychological and physiological effects
Villani et al (2007) [16]	Randomized controlled trial	34 female students and 30 male students between the ages of 21 and 28 years from the Catholic University of Milan	VR with video of tropical islands paired with audio of therapeutic sounds DVD of relaxing tropical videos Audio of therapeutic narrative	VR of a relaxing nature environment can enhance the position
Taneja et al (2017) [17]		Participants who scored 14 or less on the DASS-21^b^ test were deemed normal and eligible for the control group Participants who scored 14 or more were eligible for the stress group	SCWT^c^ task VR-based stress therapy intervention	PANAS^d^ questionnaire: a mean score of 32 for positive affect and a mean score of 15 for negative affect were generated

^a^VR: virtual reality.

^b^DASS-21: Depression Anxiety Stress Scales-21 items.

^c^SCWT: Stroop Color and Word Test.

^d^PANAS: positive and negative affect schedule.

**Table 3:** Studies on VR^a^ interventions for stress management and well-being.

Authors and year of publication	Methods	Population	Interventions	Main findings
Kaplan-Rakowski et al (2021) [18]	Between-subjects randomized design	European-based, university, business students, enrolled in an introductory computer science course, willing to participate in meditation activities	VR intervention group which used a VR headset for mediation Video control group which used a monitor for meditation	The mean differences in test scores between examinations before and after intervention were 0.03 for the VR group (SD 0.27; *P*=.01) and –0.19 for the control group (SD 0.42). VR was more effective
Hernández-Ortega et al (2021) [19]	An experimental, analytical, longitudinal, and prospective study	Participants were included if they were enrolled in Practicum I in the 2017-2018 academic year. Additionally, if they were being treated for anxiety with medication then they were excluded	Cognitive behavioral therapy (digital) Progressive muscle relaxation (digital)	Participants in IG1^b^ showed lower overall scores than those in the control group, which were also statistically significant for the KEZKAK questionnaire (*P*=.019) and state-trait anxiety (*P*=.004). Additionally, no statistical significance was found between IG1 and IG2^c^ regarding stress and anxiety
Chen et al (2012) [20]	A randomized controlled trial	Nursing students from a Spanish public university conducted clinical practices between 2017 and 2019 Inclusion criteria required enrollment in Practicum I during the 2017-2018 academic year, resulting in 59 participants. Three groups were formed: a control group (n=29), IG1 (n=15) receiving phase I, and IG2 (n=15) undergoing both phases. Students chose control or intervention groups, while random assignment determined IG1 and IG2	Digitally guided meditation sessions	SAS^d^ scores showed that postintervention participants reported lower rates of anxiety as compared to preintervention and the control group. On the other hand, SDS^e^ scores did not show a great difference between preintervention, postintervention, and the control group
Küchler et al (2020) [21]	Three-armed randomized controlled trial	College students with moderate to low mindfulness levels from universities in Germany, Austria, and Switzerland were recruited. Eligibility criteria include age 18 years or above, enrollment in university or college, proficiency in German, internet access, and moderate to low mindfulness (Freiburg Mindfulness Inventory FMI ≤ 37), with exclusion for ongoing psychotherapy or mindfulness interventions.	Studicare meditation—guided and unguided	There are no reported results at this time
Modrego-Alarcon et al (2021) [22]	Three-armed randomized controlled trial	18 years of age or older Enrollment in undergraduate or master’s degree course Enrolled in social sciences or health sciences at the Zaragoza campus or the Calatayud campus Able to speak and write in Spanish	Mindfulness-based program that consisted of 90-minute group sessions of mindfulness training Mindfulness-based program paired with short virtual reality sessions Relaxation control group	The results showed higher mean stress levels in the control group (17.73, SD 4.42) when compared to both the mindfulness-based program (15.33, SD 4.50), and the mindfulness-based program along with virtual reality (15.75, SD 4.51). VR on its own was unable to prove to be an effective intervention against stress
Berezina et al (2022) [23]	Randomized controlled trial	62 women and 24 men. Graduate students from Moscow State University of Psychology and Education. Ages 22-53 years No explicit exclusion criteria	Stimulating VR scene, followed by relaxing VR scene Relaxing VR scene, followed by stimulating VR scene Control group (no interventions)	Stimulating VR scene, then relaxing VR scene: Mean Stress (female: –1.0; *P*=.00196; male: –0.333333; *P*=.51599) Relaxing VR scene, then stimulating VR scene: Mean Stress (female: 1.900000; *P*=.00988; male: –0.500000; *P*=.58333) Control: Mean (no reported P values) Stress (female: –0.9655; male: 0.500000)

^a^VR: virtual reality.

^b^IG1: intervention group 1.

^c^IG2: intervention group 2.

^d^SAS: Self-Rating Anxiety Scale.

^e^SDS: Self-Rating Depression Scale.

## Discussion

### Measures of Well-Being Through Exposure to Nature

From the included studies that underwent data extraction, a considerable number explored the role of natural environments in a VR setting on well-being. It is well known that exposure to nature can positively improve well-being, such as daily walks in urban parks, hikes, or gardening [24]. In research led by White et al [25], it was revealed that dedicating 2 hours weekly to nature activities correlated with significantly better health and well-being outcomes compared to individuals with no nature exposure. Specifically, those who spent this time in nature had a 20% higher chance of reporting good health and a 33% higher chance of experiencing high well-being. However, access to stimulating biodiversity may not be possible for every individual, such as students in highly urbanized campuses, disabled individuals, and students who live in climates with long winter seasons. Therefore, VR may serve as an accommodation. Browning et al [11] reveal that 6 minutes of VR in an outdoor nature setting results in high positive affect levels postintervention (*P*<.001). The study emphasizes the importance of 360-degree nature videos that are immersive and interactive for adequate similarity to the natural world. O’Meara et al [10] revealed that students with high anxiety can benefit from VR nature exposure by significantly reduced negative affect (*P*=.02). The authors argue that these negative effects can be associated with examination anxiety; thus, VR serves as a well-being tool to reduce student stress.

The significance of the setting in the VR experience, namely nature, over other landscapes is explored within Valtchanov and Ellard’s [9] study. A neutral geometric environment, an urban environment, and a natural environment were randomly allocated to students with induced stress. The nature setting increased positive effects significantly (*P*<.001) from a mean of 2.21 pre-VR to 3.03 post-VR compared to no effect of the other settings. Self-reported stress significantly decreased in the natural environment (*P*<.005), unlike the geometric or urban settings [9]. Additionally, differences in the biodiversity of natural environments in VR were also compared within the included studies. Gao et al [12] conclude that despite no significant differences between the VR settings regarding impact on physiological stress, positive mood, or attention, the authors reveal that restorations in negative mood were significantly different (*P*=.03). Partly open green spaces, defined in the study as having a 10%-30% composition of trees and shrubbery in the VR environment, had the highest significant effect on reducing negative mood (at *P*<.01) [12]. To summarize, natural environments presented in the medium of VR can promote positive mood, lowered stress, and overall well-being that can supplement a lack of access that students may face. The included studies feature VR through headsets, such as the Oculus Rift. Although such devices may be cheaper than a cell phone, the average student may not want to invest in a ~US $260 headset for the purpose of accessing well-being interventions [26]. Thus, future directions of implementing VR through cell phones that can be mounted onto cheap headsets, such as the Google Cardboard paired with audio, maybe a cost-efficient but impactful VR intervention with nature for students.

### Measures of Well-Being via Stress Levels

Five of the included studies discussed interventions influencing well-being as seen through measures of stress levels. Among many therapy approaches to the reduction of stress and anxiety, relaxation techniques are seen to be very effective [17]. Specifically, the effectiveness of VR therapy was demonstrated through the use of VR environments and relaxing audio. Through the use of the PANSAS questionnaire, a mean score of 32 for positive affect was found as compared to a mean score of 15 for negative affect. Similarly, a study by Plante et al [27] looked at the impacts of VR (paired with aerobic exercise in a laboratory setting) on well-being and stress. Stress and energy were measured under 4 conditions: exercise outdoors, exercise with VR in laboratory conditions, exercise without VR in laboratory conditions, and VR without exercise. Outcomes were measured using Activation-Deactivation Adjective Checklists. It was found that exercising outdoors resulted in the most significant decreases in stress and increases in energy in men (*P*<.10) and especially in women (*P*<.50). Women also felt calmer (*P*<.50) following the VR intervention, although the findings were not significant for the men. While there is room for more research, this finding strengthens the credibility of VR as an intervention to reduce stress.

The rapid development of technology in recent years has impacted how one perceives, interprets, and organizes their day-to-day lives, which may influence one’s health and well-being [28]. When assessing well-being, an aspect that may be considered includes an individual’s sense of presence, which can be described as how one experiences and engages in events and situations at the moment [28]. Specifically, in the case of digital environments and simulations, presence can be referred to as an experience where the user is immersed in the VR environment [29]. A study conducted by Villani et al [16] found that VR intervention involving an environment with nature can help induce psychological and physiological effects such as relaxation (*P*<.005) and lowered anxiety (*P*<.01). A similar study conducted by Villani and Riva [15] reported that VR can significantly reduce anxiety levels (*P*<0.05), manage a state of anxiousness (*P*<.005), and increase relaxation (*P*<.01). Both studies used the State-Trait Anxiety Inventory to gauge anxiety levels, in addition to employing the Coping Orientation to Problems Experienced Questionnaire to evaluate participant stress levels [15,16]. In the past, researchers have suggested that a greater sense of presence can increase the level of engagement with a digital simulation [29,30]. One study conducted by Mostajeran et al [31] assessed the cognition and stress levels of office workers before and after a VR intervention involving digital plants. It was found that a digital environment with plants (SD 0.82) significantly increased the sense of presence (*P*<.05) compared to a digital environment where there were no plants (SD 0.87) [31]. Additionally, it was observed that participants performed significantly better on memory tests such as the digit span backward test (*P*<.05) after the VR intervention [31]. The findings by Mostajeran et al [31] suggest that natural VR environments can help promote a sense of presence, which can have positive effects on productivity levels, as well as mediating stress, which aligns with the findings in this study.

However, not all of the studies showed a benefit from VR. In a study from 2015, a total of 32 female undergraduate students dealing with stress and anxiety in their transition to university completed a quiet ego contemplation intervention, which reminded them of 4 characteristics of the quiet ego: detached awareness, inclusive identity, perspective taking, and growth [14]. They were split into 3 groups: an audio recording describing the 4 characteristics, the same audio recording paired with VR of a park scene, and a control group in which participants perused nature magazines. The participants completed three 15-minute sessions of their assigned condition over 6 weeks. The study found that adding the VR component to quiet ego contemplation reduced the degree to which participants felt “in the moment” (t_28_=2.66; *P*<.05). The authors hypothesized that this could be explained by the low-quality headset that was used, which was uncomfortable for participants and by the self-directed aspect of the VR, which may have been more distracting than a guided VR experience would have been.

### Measures of Well-Being via Academic Contexts

The final theme extracted from the studies included covered measures of well-being as seen in academic contexts. These studies included participants in academic institutions who are facing many stressors as a result of their academic careers. Two of those studies recruited nursing students; 1 study used cognitive behavioral therapy and progressive muscle relaxation, and the other study administered a mindfulness meditation intervention [19,20]. Ortega et al [19] used the KEZKAK questionnaire and the state-trait anxiety test as a measuring tool for stress levels. They found overall lower scores for both tests among participants who underwent the intervention with P values of .019 for the KEZKAK questionnaire and .004 for the state-trait anxiety test [19]. Similarly, Chen et al [20] used the self-rating anxiety scale as a measurement tool, where lower rates of anxiety were reported for participants in the intervention group. In considering these findings, the strength of digital-based interventions to decrease stress is further reinforced. The authors of both studies argue that these types of intervention are necessary for students to be able to function healthily and excel in their fields [19,20].

A common intervention method among these studies was the use of VR headsets. Kaplan-Rakowski et al [18] used VR headsets in their study for a meditation intervention. This was used for the experimental group, while the control group went through the intervention by watching it on a monitor. Participants went through a pretest and posttest, which entailed basic computer science tasks. It was found that the VR group (0.03) showed a higher mean difference in test scores as compared to the control group (–0.19). Berezina et al’s [23] intervention also used a VR headset. Participants in experimental group A watched a stimulating scene, followed by a relaxing scene, while participants in experimental group B watched a relaxing scene followed by a stimulating scene. Finally, there was a control group, which did not watch anything. They found that while both experimental groups experienced a decrease in fatigue, experimental group B showed a less pronounced decrease as compared to experimental group A. The final study on this theme used a VR intervention aimed at reducing stress. There were 3 groups: one which underwent a group mindfulness session, another which went through a VR session, and a control group. Modrego-Alarcón et al [22] found that participants in the mindfulness-based program showed the lowest levels of stress as compared to any other group. Additionally, the VR group scored lower stress levels compared to the control group. Overall, the studies show that VR, well-being interventions are effective in reducing stress levels and promoting relaxation among students, especially those in higher institutions.

### Strengths and Limitations

Included studies analyzed within defined categories that supported qualitative analyses of excerpts within niches of productivity that are rarely explored in demographics of young adults. This systematic review provided a diverse understanding of various techniques and research approaches to well-being in psychology in the present literature. However, given the limited research conducted on the demographic of young adults within well-being from a digital standpoint, included studies demonstrated heterogeneity, which precluded meta and statistical analyses; thus, a qualitative analysis of studies was provided. Other limitations included exclusions based on languages that were not English, which do not extend the research boundaries to non–English-speaking demographics. Additionally, given the nuance of recent developments in technology, a lack of longitudinal studies may limit the application of this systematic review. Thus, elements that may be beneficial or consequential to study participants may not be adequately observed as well-being can be a culminated outcome of long-term behaviors.

### Conclusions

In conclusion, this systematic review aimed at evaluating the efficacy of VR interventions to promote the well-being of students and young adults. To achieve this, studies were divided into themes of nature, stress, and academic contexts as focuses of interventions. Overall, the included studies reveal that VR interventions pose a promising medium to reduce the stress experienced by young adults and students, which can ultimately improve well-being. These findings reveal that VR may serve as an accessible, affordable tool for students and young adults to promote well-being or lower stress levels. However, there are some limitations to the review. The included studies tend to have smaller sample sizes, which may not be representative of students as a whole. A total of 20 studies were included in the final phase of extraction. Future directions may include expanding the search criteria to include more studies that may have higher sample sizes.

## Appendix

Multimedia Appendix 1 PRISMA 2020 checklist.

## Abbreviations

AMSTAR-2: Assessing the Methodological Quality of Systematic Reviews
GRADE: Grading of Recommendations Assessment, Development, and Evaluation
MBI: mindfulness-based intervention
PRISMA-P: Preferred Reporting Items for Systematic Review and Meta-Analysis Protocol
VR: virtual reality
